# Most women recover from psychological distress after postoperative complications following implant or DIEP flap breast reconstruction: A prospective long-term follow-up study

**DOI:** 10.1371/journal.pone.0174455

**Published:** 2017-03-27

**Authors:** Reinier Timman, Jessica P. Gopie, J. Nick Brinkman, Annelies Kleijne, Caroline Seynaeve, Marian B. E. Menke-Pluymers, Moniek M. ter Kuile, Aad Tibben, Marc A. M. Mureau

**Affiliations:** 1 Department of Psychiatry, section of Medical Psychology and Psychotherapy, Erasmus MC, University Medical Center Rotterdam, Rotterdam, The Netherlands; 2 Department of Clinical Genetics, Leiden University Medical Center, Leiden, The Netherlands; 3 Department of Plastic and Reconstructive Surgery, Erasmus MC Cancer Institute, University Medical Center Rotterdam, Rotterdam, the Netherlands; 4 Department of Medical Oncology, Erasmus MC Cancer Institute, University Medical Center Rotterdam, Rotterdam, the Netherlands; 5 Department of Surgical Oncology, Erasmus MC Cancer Institute, University Medical Center Rotterdam, Rotterdam, the Netherlands; 6 Breast Clinic, Albert Schweitzer Hospital, Rotterdam, the Netherlands; 7 Department of Gynecology, Leiden University Medical Center, Leiden, The Netherlands; University of Rochester, UNITED STATES

## Abstract

**Background:**

Substantial complication rates after postmastectomy breast reconstruction (BR) in breast cancer patients have been reported. Few studies have reported on the resulting psychological distress (PD) and satisfaction with the aesthetic result in relation to postoperative complications after completion of implant or DIEP flap BR. The present study investigated whether women were able to recover from complication related distress in the long term.

**Methods:**

PD was prospectively measured using questionnaires regarding anxiety, depression and cancer distress. Eligible patients completed questionnaires before BR (T0, *n* = 144), after one month (T1, *n* = 139) and after completion of BR, approximately 21 months after initial reconstructive surgery (T2, *n* = 119). Satisfaction with the aesthetic result was assessed 21 months after BR. Data concerning complications, subsequent additional surgery and total reconstruction failure up to T2 were collected from the medical records. Analyses were performed using multi-level regression analyses correcting for age.

**Results:**

One or more complications occurred in 61 patients (42%) and 50 women required subsequent surgery (35%). In time, mean PD significantly declined towards baseline scores independent of complications. However, a total reconstruction failure (*n* = 10) was significantly associated with a large temporary increase in depression scores. After additional surgery due to complications patients were less satisfied with aesthetic outcome, although patient satisfaction was independent of PD.

**Conclusions:**

PD outcomes generally declined to normal levels after completion of the entire BR course. Patients experiencing a total reconstruction failure reported more depression after this loss, but in the long term recovered to the same level as women without complications. These findings indicate that women generally can cope efficiently with these serious adverse events, even if they were less satisfied with the aesthetic result.

## Introduction

Breast reconstruction (BR) after mastectomy for breast cancer is generally a well considered option in case of either therapeutic treatment or preventive surgery. BR is intended to improve body image and quality of life, however, there is a considerable risk of complications which can lead to adverse psychosocial effects [1,2,3,4]. Understandably, BR will be accompanied by psychological distress but it may be expected that, in the longer term, women with adequate psychosocial resources will recover from distress, even after experiencing complications or less satisfaction with aesthetic outcome than expected. Yet, clinical experience shows that a sub-group of women remains distressed and finds it difficult to resume their lives in the longer term. Identification of risk factors for psychological distress after BR, including complications and subsequent additional surgery, allows professionals to adequately inform their patients, and to offer support if necessary, in addition to routine medical care.

Complications following implant BR are for example infection, hematoma, seroma, and implant removal [4,5]. After autologous BR (e.g., DIEP flap) hematoma, seroma, partial and total flap loss may occur [6,7,8]. Previous studies demonstrated that undergoing BR can be experienced as a difficult and complicated process with an unexpectedly long recovery period and at the cost of additional scarring at the recipient and/or donor-site [9,10,11,12].

Although many studies investigated the psychosocial impact of BR after mastectomy, only few, mostly retrospective studies, focused on the effects of *complications* and subsequent surgery on psychological distress [10,13,14,15,16]. One retrospective quantitative study (*n* = 60) covering a very short follow-up period of three months after surgery, reported that psychological distress levels were similar for women with and without complications [14]. Two qualitative studies in very small samples (*n* = 6, and *n* = 21) found that women were unprepared for the BR course, that they felt it was burdensome physically as well as emotionally, and that the additional operations and the long recovery period were disappointing and unexpected, regardless of complications [10,15]. A recent prospective study covering a follow-up period of more than one year (n = 97) demonstrated a significant impact of complications on psychological wellbeing after autologous BR [16].

In our previous prospective study on the *short-term* impact of complications after BR we found that women experiencing complications reported significantly higher levels of psychological distress one month after the first reconstructive intervention [13]. The present *long-term* prospective follow-up study aimed at investigating the relationship of post BR complications with psychological distress after completion of the entire BR course using an implant or DIEP flap. We hypothesized that in the long-term psychological distress will return to preoperative levels independent of the occurrence of complications, subsequent additional surgery and satisfaction with the end result [9,10,11,12,16].

## Materials and methods

### Patients

The current study is part of a multi-center prospective follow-up investigation on the psychosocial impact of BR after either prophylactic or therapeutic mastectomy. Participants for the current study were women who opted for BR with either an implant or a DIEP flap after mastectomy for breast cancer. Exclusion criteria were: (unsuccessful) BR in the past, detection of recurrent or metastatic breast cancer either before or during study follow-up, and not being able to understand and speak the Dutch language sufficiently. Women who did not consent or who did not respond to the primary and the reminder invitation were considered as non-respondents. Patients were approached between December 2007 and May 2010 at the Leiden University Medical Center (LUMC); Erasmus MC, University Medical Center Rotterdam; Haga Teaching Hospital the Hague; Rijnland Hospital Leiderdorp; Lange Land Hospital Zoetermeer; Admiral the Ruyter Hospital (Goes, Vlissingen); and Hospital ZorgSaam Zeeuws-Vlaanderen, the Netherlands. Ethics approval was obtained from all participating hospitals.

In total, 196 breast cancer patients awaiting BR were invited for the study of whom 151 consented to participate. Seven women were excluded from further analyses due to recurrent disease leaving data from 144 patients suitable for analysis (69 implant BR; 75 DIEP flap BR).

### Procedure

Before surgery, an invitation letter explaining the procedure and purpose of the study, an informed consent, and a prepaid envelope were sent to all women on the BR waiting lists of the participating hospitals. A reminder was sent by letter if patients did not respond within two weeks or they were contacted by phone if surgery was planned on the short-term. Patients who consented to participate received a questionnaire including a range of demographic, clinical and psychosocial items which they were requested to fill in before the breast reconstructive surgery (T0). Similar questionnaires were asked to fill in one month after this surgery (T1), and at the end of the complete BR course (T2). Additional questions at T2 concerned (type of) complications after BR, additional subsequent surgical interventions and total reconstruction failure.

### Questionnaires

#### Dependent variables

The term “psychological distress” (PD) is used as a general term in this paper covering the concepts anxiety, depression and cancer distress.

***Anxiety and depression*.** Anxiety and depressive symptoms were measured with the Hospital Anxiety and Depression Scale (HADS) [17]. The HADS consists of 14 items (S1 Appendix) rated on a 4-point Likert scale and includes two subscales, measuring anxiety and depression (both 7 items). Both subscale scores range from 0 to 21. A score of 8 or above can be used as a cut-off for clinical significance [17,18]. Good reliability and validity have been reported for the HADS [18,19]. Cronbach's alpha in our sample was 0.87 for anxiety and 0.86 for depression.

***Breast cancer specific distress*.** Cancer-specific distress regarding breast cancer was measured using the Impact of Event Scale (IES) [20,21]. The IES consists of 15 items (S2 Appendix) that are rated on a 4-point Likert scale. The total IES score (range 0–75) measures the extent to which one is overwhelmed by intrusive thoughts and avoidant behavior regarding a specific traumatic event, in this case ‘breast cancer’. A cut-off score of 20 or can be used as an indication for high symptom levels [22]. Reported reliability and validity of the IES are satisfactory [20,21,22,23] and in our sample Cronbach's alpha was 0.88.

***Patient satisfaction*.** At T2 patient satisfaction with the aesthetic outcome was rated on a 10-point scale as used in a previous study, ranging from 1 (extremely dissatisfied) to 10 (extremely satisfied) [24].

#### Background variables

***Baseline characteristics***. At T0, baseline demographic characteristics (e.g., age, having a partner or children, educational level) were collected with questionnaires. Clinical data (e.g., body mass index (BMI), adjuvant therapy, type of BR) were collected from the medical files. If the exact date of breast cancer diagnosis was unclear, the date of mastectomy was used.

***Postoperative complications and subsequent surgery*.** At T2, after completion of the entire BR course, the occurrence of postoperative complications and subsequent surgery up to T2 were collected from the medical records (JPNG, MAMM, JNB). A complication was defined as any adverse physical event specifically related to BR occurring until the T2 assessment. A major event was defined as a complication leading to subsequent surgery, not including surgery for aesthetic improvements or the exchange of tissue expanders with implants. Furthermore, a total reconstruction failure, assumed to be of major impact as well, was defined as: loss of tissue expander or implant, or total flap necrosis which was not salvaged with a new BR within the current study period.

### Statistical analyses

Descriptive statistics were calculated for all variables. Baseline differences between participants and women loss to follow-up were analyzed by using chi-square tests corrected for continuity. Mann-Whitney U tests were used to investigate the relationship between patient satisfaction scores and the occurrence of complications, subsequent surgery, and total reconstruction failure. Spearman's rank correlations were applied for the relation between patient satisfaction and PD. Chi square tests were used to analyze the difference between the types of BR technique regarding complications and subsequent surgery, number of re-operations, and total reconstruction failure.

For outcome measures, missing individual items from a subscale were inferred by using the mean of the remaining items, if at least 70% of all items were completed. To investigate changes in time for anxiety, depression and cancer distress, multi-level regression analyses (MLA) were performed, which can efficiently handle incomplete time-series data with a minimal loss of information, and correct for bias when absence of data is dependent on characteristics that are present in the models [25]. Potential predictors of distress were additional surgery and total reconstruction failure.

We corrected for relevant background variables (covariates radiotherapy, type of BR and age at BR).

For the dependent variables anxiety, depression and cancer distress, models were postulated including linear and quadratic time effects and the interactions with the eligible predictors and covariates in the regression models. The deviance statistic [26] using restricted maximum likelihood [27] was applied to determine an appropriate covariance structure. Estimates at T0, T1 and T2 from the MLA models were calculated, as well effect sizes of change since baseline. Additional time dependent effects of significant covariates were calculated.

Because three primary outcomes were analysed, we applied a Bonferroni correction and considered a two-sided *p*-values < 0.0167 as statistically significant. Data were analysed with the statistical package SPSS 21.0 (IBM-SPSS Inc., Chicago).

## Results

### Patient samples

Seven women were excluded from the analyses as they developed recurrent or metastatic breast cancer after completion of the first questionnaire Furthermore, 25 women were lost during follow-up as five patients did not complete the assessment at T1, and 25 women did not complete the T2 assessment. However, their available data from other time points were included in the analyses. Reasons for loss to follow-up were; one patient with diabetes mellitus died 18 days after DIEP flap BR due to pneumonia and sepsis, eight women stopped participation and 16 persons did not respond at all. The total number of dropouts at T2 (*n* = 25) did not differ significantly from participating patients at T2 (*n* = 119) regarding demographic variables, additional surgery for complications, total reconstruction failure, and baseline anxiety, depression and cancer distress score (data not shown). Of note, a larger proportion of dropouts had complications (64% vs. 38%, *p* = 0.03).

Patient characteristics and post-BR complications are reported for all participants who met the inclusion criteria and who completed T0 (*n* = 144, Tables 1 and 2). Further analyses were performed on baseline as well as follow-up assessments (T0: *n* = 144; T1: *n* = 139; T2: *n* = 119, Table 3).

**Table 1:** Baseline patient characteristics of 144 women undergoing breast reconstruction after therapeutic mastectomy.

	Mean (sd)
Age in years at time of BR	49.4 (8.7)
Time since mastectomy in years	2.3 (3.3)
BMI in kg/m^2^	25.9 (4.2)
	N (%)
Having a partner	120 (83.3)
Having children	126 (87.5)
Education level	
Low	26 (18.1)
Intermediate	55 (38.2)
High	63 (43.8)
Inherited predisposition for BC ^a^	38 (26.4)
Laterality	
Unilateral BR	108 (75.0)
Bilateral BR	36 (25.0)
Timing of BR	
Immediate BR	46 (31.9)
Delayed BR	98 (68.1)
BR type	
Implant BR	69 (47.9)
DIEP-flap BR	75 (52.1)
Therapies	
Radiation therapy	80 (55.6)
Chemotherapy	41 (28.5)
Hormonal therapy	61 (42.4)

^a^: brca1/brca2/familial risk.

BC: breast cancer; BMI: body mass index; DIEP: Deep Inferior Epigastric artery Perforator; BR: breast reconstruction; sd: standard deviation.

**Table 2:** Postoperative complications after implant and DIEP flap BR.

	Implant BR	DIEP flap BR	*P*-value*
	N = 69	N = 75	
	N (%)	N (%)	
One or more complications	31 (44.9)	30 (40.0)	0.67
Additional surgery for complications	28 (40.6)	22 (29.3)	0.22
More than one additional operation	14 (20.3)	8 (10.7)	0.17
Total reconstruction failure	9 (13.0)	1 (1.3)	0.01
Type of complications			
*Wound healing complications*			
Wound dehiscence	1 (1.4)	0	0.97
Wound infection	16 (23.2)	2 (2.7)	0.001
Hemorrhage leading to surgery	3 (4.3)	2 (2.7)	0.92
Hematoma	2 (2.9)	7 (9.3)	0.21
Partial mastectomy skin flap necrosis	1 (1.4)	3 (4.0)	0.67
Seroma	0	2 (2.7)	0.51
Abscess	1 (1.4)	1 (1.3)	1.00
*Implant-related complications*			
Prosthesis malposition	2 (2.9)		
Implant or tissue expander perforation	2 (2.9)		
Capsular contracture	4 (5.7)		
Definite loss of implant/expander	9 (13.0)		
*DIEP Flap-related complications*			
Fat necrosis		7 (9.3)	
Venous congestion		1 (1.3)	
Partial flap necrosis		2 (2.7)	
Total flap loss		1 (1.3)	
Abdominal wound healing problems		4 (5.3)	
Abdominal herniation		1 (1.3)	
*General complications*			
Radiodermatitis	1 (1.4)	0	0.97
Pneumothorax	1 (1.4)	0	0.97
Symptomatic pulmonary embolism	0	1 (1.3)	1.00
Subcutaneous extravasation i.v. line	0	2 (2.7)	0.51

DIEP: Deep Inferior Epigastric artery Perforator; BR: breast reconstruction.

* Chi^2^ test with correction for continuity.

**Table 3:** Estimates of psychological distress in time and effects of additional surgery and reconstruction failure.

	Anxiety ^2)^			Depression ^2)^			Cancer distress ^3)^		
No covariate effects ^1)^	Estimate	*d*	*P-value*	Estimate	*d*	*P-value*	Estimate	*d*	*P-value*
Baseline	5.3			5.3			24.2		
1 month	3.8	- 0.43	0.009	5.3	-0.01	0.953	16.1	- 0.65	<0.001
21 months	3.6	- 0.49	0.004	4.7	- 0.16	0.372	15.2	- 0.72	<0.001
Additional effects		^4)^			^4)^			^4)^	
Additional surgery									
Baseline	5.2	- 0.04	0.856	5.3	- 0.01	0.948	23.9	- 0.03	0.879
1 month	5.0	0.35	0.077	5.9	0.16	0.410	16.9	0.06	0.743
21 months	2.3	- 0.36	0.082	3.3	- 0.34	0.097	13.7	- 0.12	0.553
Total reconstruction failure									
Baseline	7.0	0.46	0.177	6.0	0.17	0.622	30.8	0.53	0.136
1 month	5.3	0.42	0.217	9.2	0.96	0.006	23.3	0.58	0.090
21 months	4.5	0.28	0.473	5.1	0.09	0.812	20.4	0.42	0.272
10 years younger									
Baseline	6.5	0.34	0.001	6.2	0.20	0.055	25.9	0.13	0.215
1 month	5.0	0.33	0.002	6.5	0.30	0.005	20.6	0.36	0.001
21 months	4.5	0.27	0.015	5.4	0.16	0.146	19.8	0.37	0.001

^1)^ At mean age, without complications.

^2)^ HADS: Hospital Anxiety and Depression Scale (0–21), higher scores indicate higher anxiety and depression levels.

^3)^ IES: Impact of Event Scale (range 0–75), higher scores indicate higher distress levels.

^4)^ Cohen's *d*, effect size compared to estimates without covariate effects.

Two-sided p-values of < 0.0167 are considered significant.

### Demographic and clinical characteristics

Baseline patient characteristics are shown in Table 1. The time period between therapeutic mastectomy and BR was on average 2.3 years, (*sd* = 3.3, median = 1.6 yrs, range = 0–20.3 yrs). Questionnaires at T2 were completed on average 21.4 months after the first reconstructive intervention (*sd* = 5.9 months, median = 20.4 months, range = 8.9–37.7 months).

### Postoperative complications and subsequent surgery

Complications after BR, subsequent surgery for complications and total reconstruction failure are described in Table 2. During the entire BR course, 61 patients (42%) experienced one or more complications (e.g., wound infection, skin necrosis, hematoma or wound dehiscence) and 50 patients (35%) of the total sample consequently needed additional surgery. Twenty-two women (15%) underwent more than one additional surgical intervention due to complications (range: 2–5). Reasons for additional surgery were for example: hematoma drainage, replacement of an infected tissue expander, or microvascular revision. Ten women (6.9%) had a total reconstruction failure during the follow-up period, occurring in significantly more patients with an implant BR (*n* = 9) versus a DIEP flap BR (*n* = 1; *p* = 0.01). There were no differences in the occurrence of complications or (more than one) additional surgical interventions between women with either implant or DIEP flap BR (*p* = 0.67 and *p =* 0.22, respectively). Significantly more wound infections occurred after implant BR (*p =* 0.001, see Table 2).

### Potential predictors and covariates

Complications in general (n = 61) had a too high co-incidence with complications followed by additional surgery (n = 50) and could not be included in the multivariate MLA because it caused problems of multi-collinearity. Therefore, additional surgery and a total reconstruction failure were used as predictors in the MLA analyses.

### The general course of psychological and cancer distress

In S3 Appendix, the estimated parameters of the MLA analyses are presented. The combination of the linear and quadratic effects is not directly insightful. For an easy interpretation the resulting estimations on the three time points and the differences are shown in Table 3. For women without complications and at mean age (upper rows), anxiety significantly declined after surgery (with Cohen's effect sizes of *d* = -0.43 from T0 to T1 and *d* = -0.49 from T0 to T2). Cancer distress significantly decreased from T0 to T1 (*d* = -0.65) and from T0 to T2 (*d* = -0.72). No change over time was observed in depression scores.

### The impact of reconstruction failure and additional surgery on psychological distress after breast reconstruction

A total reconstruction failure was significantly related to a large temporary increase of depression scores at T1 (*d* = 0.96, *p* = 0.006). At T2, the estimated depression scores returned to levels comparable to women without a reconstruction failure (*d* = 0.09, *p* = 0.81). One woman, with a baseline depression score of 10, had a higher score (17) at the long term follow-up. Additional surgery was not significantly related to anxiety, depression or cancer distress (Table 3).

In addition, the MLA model demonstrated covariate effects for age with anxiety, depression, and cancer distress at several time points, indicating that younger women had higher anxiety scores during the entire BR course, higher depression scores one month after the reconstruction and higher cancer distress scores one and 21 months after BR. Women with a DIEP flap BR had lower cancer distress scores than women with an implant BR during the entire BR course.

### Patient satisfaction

Satisfaction with aesthetic outcome was rated by 114 patients at T2, yielding a mean score of 7.68 (*sd* = 1.41, median 8.00, range 2–10). Women who underwent additional surgery due to complications were less satisfied with the aesthetic result (*M* = 7.23) than women who had not undergone additional surgery (*M* = 7.84; Mann-Whitney U test, *U* = 866.5, *p* = 0.005). In addition, women with an implant BR were less satisfied (*M* = 7.16) than women with a DIEP flap BR (*M* = 8.10; Mann-Whitney U test, *U* = 1054.0, *p* = 0.001). No significant correlations were observed with anxiety (r = -0.024; p = 0.803), depression (r = -0.089; p = 0.360) and cancer distress (r = -0.016; p = 0.872) 21 months after BR.

## Discussion

The present study prospectively investigated the relationship between complications, subsequent additional surgery as well as a total reconstruction failure with psychological distress and patient satisfaction, before and after completion of breast reconstruction (BR) with either an implant or DIEP flap. The overall complication rate after BR in this study was comparable to data of other studies [14,28,29]. As expected, our study confirmed our hypothesis (and clinical experience) as well as Lu’s findings [16] and our clinical experience that in the long term most women recover from psychological distress. Understandably, the occurrence of complications leading to subsequent surgery was associated with lower patient satisfaction with aesthetic outcome.

Women who had to face a total reconstruction failure reported higher depression levels at the short-term follow-up assessment. Six of these ten women had depression scores in the clinically relevant range, illustrating the distressing period after realizing that the reconstructed breast could not be salvaged. Only one of them, who also had a depression score in the clinical range at baseline, still had such a score at the long-term follow-up.

The finding that DIEP flap BR patients were more satisfied with aesthetic outcome than implant BR patients corroborates results from previous studies [24,30,31,32,33,34]. However, the reported differences could also have been caused by the higher proportion of reconstruction failures in implant compared to DIEP flap BR patients [35]. An indication for this explanation was also provided by Yang et al. who reported no differences in satisfaction between the various types two years after the reconstruction and noted that complications played a significant role [36]. A finding that was not replicated by Zhang et al. who reported a non-significant relation between complications and satisfaction [37]. During the entire BR course DIEP flap BR patients reported lower anxiety distress scores than implant BR patients, this could be caused by the longer time between mastectomy and BR [38]. This longer period may have provided the opportunity to cope better with the intruding situation.

Complications after other types of BR, such as the TRAM flap, were not investigated in the present study, but are also an important area of future research. Nevertheless, we feel that the findings from the current prospective study with a sufficiently large patient group are important to incorporate in the information provision and referral of breast cancer patients considering breast reconstruction. Patients should be properly informed on the risks of complications and additional surgery, total reconstruction failure, accompanying psychological distress, and the potential of psychological adjustment. They should be counseled to adopt realistic expectations. Patient-centred care, focussing on adequate preoperative information and the quality of the relationship between patient and surgeon can increase patient satisfaction [39, 40].

In conclusion, mean scores of psychological distress returned to normal levels after completion of the entire BR course, independent of a lower patient reported satisfaction after complications. Patients experiencing a total reconstruction failure reported temporarily more depression directly after the loss of their neo-breast.

## Supporting information

S1 Appendix Item wording of the Hospital Anxiety and Depression Scale. (PDF)

S2 Appendix Item wording of the Impact of Event Scale. (PDF)

S3 Appendix Multilevel models with additional surgery and total reconstruction failure as predictors of psychological distress in women undergoing BR and the covariates reconstruction type, radio therapy and age. (PDF)

S1 File SPSS syntax file. (SPS)

S2 File SPSS data file. (SAV)

S3 File Excel additional calculation file. (XLS)
