# Supplementary material for: Most women recover from psychological distress after postoperative complications following implant or DIEP flap breast reconstruction: A prospective long-term follow-up study
Source: PLoS One. 2017 Mar 27;12(3):e0174455. doi: 10.1371/journal.pone.0174455 (PMC5367706; doi:10.1371/journal.pone.0174455)
Supplement: S1 Appendix — (PDF) [file pone.0174455.s001.pdf]

**S1 Appendix.** Item wording of the Hospital Anxiety and Depression Scale.

| | | |
|---|---|---|
| 1. | I feel tense or 'wound up'. | (A) |
| 2. | I still enjoy the things I used to enjoy. | (D) |
| 3. | I get a sort of frightened feeling as if something awful is about to happen. | (A) |
| 4. | I can laugh and see the funny side of things. | (D) |
| 5. | Worrying thoughts go through my mind. | (A) |
| 6. | I feel cheerful. | (D) |
| 7. | I can sit at ease and feel relaxed. | (A) |
| 8. | I feel as if I am slowed down. | (D) |
| 9. | I get a sort of frightened feeling like 'butterflies' in the stomach. | (A) |
| 10. | I have lost interest in my appearance. | (D) |
| 11. | I feel restless as I have to be on the move. | (A) |
| 12. | I look forward with enjoyment to things. | (D) |
| 13. | I get sudden feelings of panic. | (A) |
| 14. | I can enjoy a good book or radio or TV program. | (D) |

Zigmond AS & Snaith RP. The hospital anxiety and depression scale. Acta Psychiat Scand. 1983;67(6), 361-370.
