# Supplementary material for: Most women recover from psychological distress after postoperative complications following implant or DIEP flap breast reconstruction: A prospective long-term follow-up study
Source: PLoS One. 2017 Mar 27;12(3):e0174455. doi: 10.1371/journal.pone.0174455 (PMC5367706; doi:10.1371/journal.pone.0174455)
Supplement: S2 Appendix — (PDF) [file pone.0174455.s002.pdf]

S2 Appendix. Wording of the Impact of Event Scale.

1. I thought about breast cancer when I didn't mean to.
2. I avoided letting myself get upset when I thought about it or was reminded of it.
3. I tried to remove breast cancer from my memory.
4. I had trouble falling asleep or staying asleep, because of pictures or thoughts about breast cancer that came into my mind.
5. I had waves of strong feelings about it.
6. I had dreams about breast cancer.
7. I stayed away from reminders of breast cancer.
8. I felt as if it hadn't happened or it wasn't real.
9. I tried not to talk about breast cancer.
10. Pictures about breast cancer popped into my mind.
11. Other things kept making me think about breast cancer.
12. I was aware that I still had a lot of feelings about it, but I didn't deal with them.
13. I tried not to think about breast cancer.
14. Any reminder brought back feelings about it.
15. My feelings about it were kind of numb.

Horowitz MJ, Wilner N & Alvarez W. Impact of event scale: a measure of subjective stress. Psychosom Med. 1979;41, 209-218.
