# Supplementary material for: Most women recover from psychological distress after postoperative complications following implant or DIEP flap breast reconstruction: A prospective long-term follow-up study
Source: PLoS One. 2017 Mar 27;12(3):e0174455. doi: 10.1371/journal.pone.0174455 (PMC5367706; doi:10.1371/journal.pone.0174455)
Supplement: S3 Appendix — (PDF) [file pone.0174455.s003.pdf]

S3 Appendix. Multilevel models with additional surgery and total reconstruction failure as predictors of psychological distress in women undergoing BR and the covariates reconstruction type, radio therapy and age.

	Intercept		time linear		time quadratic	
	estimate	p-value	estimate	p-value	estimate	p-value
**Anxiety**						
No covariate effects	5.31	<.001	-1.58	.010	0.07	.012
Additional surgery	-0.12	.856	1.43	.047	-0.07	.036
Total reconstruction failure	1.65	.177	-0.14	.909	0.01	.927
DIEP vs. implantation	-0.84	.184	0.35	.593	-0.01	.647
Radio therapy	-0.06	.933	0.58	.406	-0.03	.389
Age	-0.12	.001	0.01	.887	0.00	.908
**Depression**						
No covariate effects	5.32	<.001	-0.04	.955	0.00	.988
Additional surgery	-0.05	.948	0.76	.376	-0.04	.327
Total reconstruction failure	0.70	.622	3.43	.023	-0.16	.020
DIEP vs. implantation	-0.16	.825	0.59	.455	-0.03	.459
Radio therapy	-0.08	.914	0.52	.530	-0.02	.529
Age	-0.08	.055	-0.04	.355	0.00	.336
**Cancer distress**						
No covariate effects	24.23	<.001	-8.55	<.001	0.39	<.001
Additional surgery	-0.37	.879	1.22	.570	-0.06	.545
Total reconstruction failure	6.58	.136	0.71	.855	-0.04	.838
DIEP vs. implantation	-5.77	.010	2.73	.169	-0.12	.205
Radio therapy	1.92	.408	2.22	.286	-0.11	.265
Age	-0.16	.215	-0.30	.011	0.01	.013
